# Growth selectivity control of InAs shells on crystal phase engineered GaAs nanowires†

**DOI:** 10.1039/d2na00109h

**Published:** 2022-04-08

**Authors:** Víctor J. Gómez, Mikelis Marnauza, Kimberly A. Dick, Sebastian Lehmann

**Affiliations:** Nanophotonics Technology Center, Universidad Politécnica de Valencia Camino de Vera, s/n Building 8F | 2^a^ Floor 46022 Valencia Spain vjgomher@ntc.upv.es; Solid State Physics and NanoLund, Lund University Box 118 S-221 00 Lund Sweden Sebastian.Lehmann@ftf.lth.se; Centre for Analysis and Synthesis and NanoLund, Lund University Box 124 221 00 Lund Sweden

## Abstract

In this work we demonstrate a two-fold selectivity control of InAs shells grown on crystal phase and morphology engineered GaAs nanowire (NW) core templates. This selectivity occurs driven by differences in surface energies of the NW core facets. The occurrence of the different facets itself is controlled by either forming different crystal phases or additional tuning of the core NW morphology. First, in order to study the crystal phase selectivity, GaAs NW cores with an engineered crystal phase in the axial direction were employed. A crystal phase selective growth of InAs on GaAs was found for high growth rates and short growth times. Secondly, the facet-dependant selectivity of InAs growth was studied on crystal phase controlled GaAs cores which were additionally morphology-tuned by homoepitaxial overgrowth. Following this route, the original hexagonal cores with {110} sidewalls were converted into triangular truncated NWs with ridges and predominantly {112}_B_ facets. By precisely tuning the growth parameters, the growth of InAs is promoted over the ridges and reduced over the {112}_B_ facets with indications of also preserving the crystal phase selectivity. In all cases (different crystal phase and facet termination), selectivity is lost for extended growth times, thus, limiting the total thickness of the shell grown under selective conditions. To overcome this issue we propose a 2-step growth approach, combining a high growth rate step followed by a low growth rate step. The control over the thickness of the InAs shells while maintaining the selectivity is demonstrated by means of a detailed transmission electron microscopy analysis. This proposed 2-step growth approach enables new functionalities in 1-D structures formed by using bottom-up techniques, with a high degree of control over shell thickness and deposition selectivity.

## Abstract 2

Growth rate and growth time are the two key factors governing the crystal phase and facet dependent selective growth in the GaAs/InAs core/shell system. In addition, we demonstrate control over the shell thickness and selectivity.

## Graphical abstract

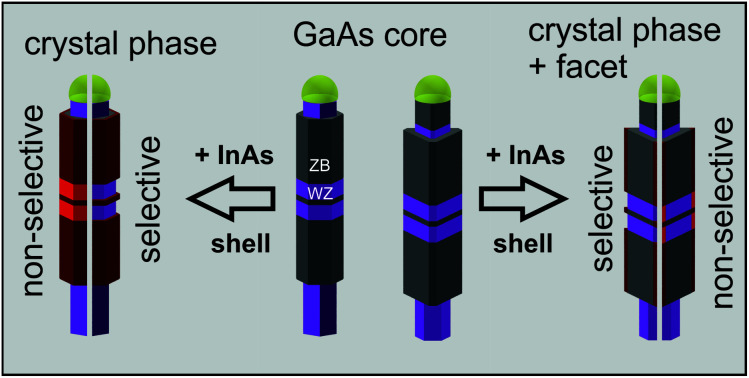

## 1. Introduction

While coherent epilayer growth in planar heterostructures is highly restricted to a narrow lattice parameter window, nanoscale structures, such as nanowires (NWs), allow heterostructures to overcome the limits regarding lattice mismatch and strain relief. In general, the preferred growth mode of a heterostructure is specific to the surface, interface, and lattice mismatch (thermodynamic constraints), which are in turn determined by the choice of substrate and epilayer. Despite being specific to the combination of substrate and epilayer, these thermodynamic constraints can be overcome kinetically to some extent, by either adjusting epitaxial growth conditions,^1^ by changing the surface chemistry or by using surfactant elements.^2–6^ In this way *e.g.*, 3D Stranski–Krastanov (SK) island formation^7^ can be kinetically hindered in favor of a two-dimensional (2D) Frank–van der Merwe (FM) growth mode and *vice versa*. The SK growth of quantum dots (QDs) in the (In, Ga)As/GaAs material system is widely studied for its single photon emission properties.^8^ While SK growth is favored on GaAs(100), the 2D FM mode occurs on other low-index GaAs surfaces such as (110) or (111) and misfit strain relaxes plastically.^9–12^

Heterostructured NWs enable the combination of different materials with resulting minimal strain compared to their planar counterparts.^13,14^ These heterostructures can be grown either in the axial or in the radial direction (core–shell approach) with a high degree of control over interfaces and composition.^15–18^ Novel device architectures can be realized based on pioneering material combinations, otherwise highly defective in their planar form. Studies of strain relaxation in core–shell NW heterostructures disclose that plastic relaxation is suppressed compared to their planar counterparts.^14,19–21^ Nonetheless, the formation of misfit dislocations,^21,22^ QDs,^23–26^ and shell surface roughening^27–29^ has been demonstrated for very high mismatch.

Previous works on the strain relaxation in core–shell NWs have focused mainly on varying NW dimensions for fixed lattice mismatch (*e.g.*, Si/Ge or GaAs/InAs), where GaAs NWs often present {110} facets, favored by their lower surface energy, and the growth of QDs on these structures requires the use of foreign materials such as Bi.^30,31^ A promising facet dependent selective growth (the preferential shell growth on one facet over the other) has been demonstrated for Si/Ge NWs,^32,33^ GaAs/InAs NWs,^34^ and NW networks.^35^

In addition, it has been demonstrated that III–V NW growth can be tuned to exhibit different lateral facet terminations.^36–39^ Furthermore, crystal phase selective core–shell growth is demonstrated for different core–shell combinations, such as GaAs/InAs,^40^ InAs/GaSb,^41,42^ and InAs/AlSb,^43^ where the selectivity is enabled by the difference in surface energies between the wurtzite (WZ) and zinc blende (ZB) polytypes. However, despite all the mentioned selectivity examples, and due to the exponential complexity increase of core–shell systems when different materials, crystal phases, and facet terminations are involved, there is a lack of control over the factors governing the selective growth and over the energetically preferred growth mode.

**Fig. 1:**
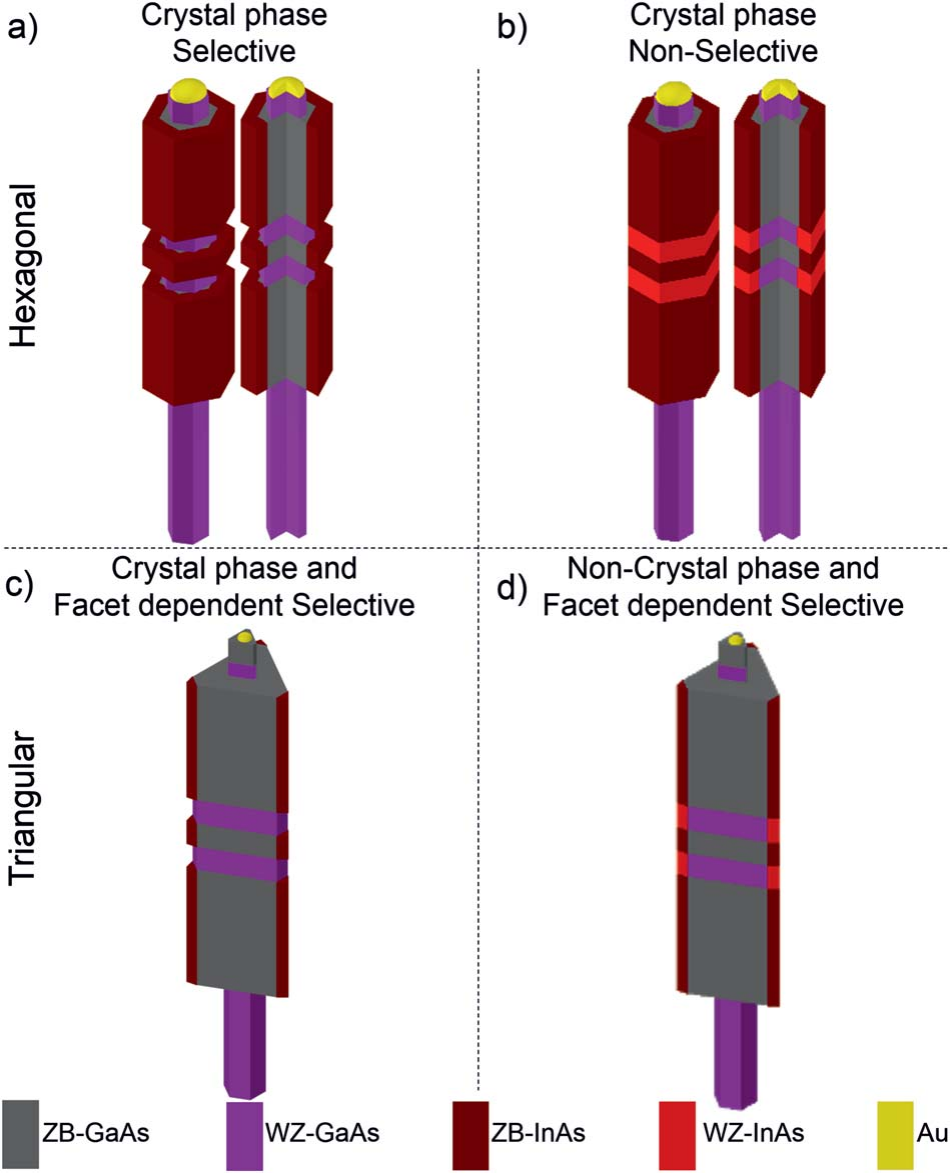
Schematic representation of the hexagonal and triangular truncated GaAs NW cores and InAs shells for different selectivity conditions.

In this work we demonstrate control over the crystal phase and facet dependent selectivity in the GaAs/InAs core–shell growth (Fig. 1) and we identify the two key growth parameters, growth rate and growth time, governing the selective core–shell growth. The observed selectivity occurs either due to the difference in crystal phase (Fig. 1a), due to the different lateral facet termination present in the NW core (Fig. 1d), or due to both factors (Fig. 1c). Firstly, to study the crystal phase selectivity mechanism, GaAs NW cores with controlled crystal structure along the axial direction were employed. A crystal phase selective growth of InAs on GaAs was found for high growth rates and short growth times. However, for extended growth times selectivity is lost, *i.e.* the InAs shell homogeneously covers the entire GaAs core (Fig. 1b). Secondly, the facet-dependant selectivity mechanism was studied on morphology-tuned GaAs cores with triangular truncated cross-section, consisting of crystal phase-controlled GaAs NWs, which were homoepitaxially overgrown. By precisely controlling the conditions of InAs heteroepitaxial overgrowth, the growth of InAs can be promoted over the ridges and reduced over the {112}_B_ facets (Fig. 1d) and even, additionally maintain the crystal phase selectivity (Fig. 1c). However, the two types of selectivity studied (crystal phase- and facet termination selective growth) are lost when the growth time is above a critical limit, thus, restricting the total thickness of the shell grown under conditions promoting the selectivity. To keep the mentioned selectivity, we propose a 2-step growth process combining a high with a low growth rate step. We demonstrate, by means of a detailed high resolution transmission electron microscopy (HR-TEM) analysis, control over the thickness of the InAs shells while maintaining the selectivity of the growth.

## 2. Experimental

All studied samples were grown on commercially available, double-side polished, epiready GaAs(111)_B_ substrates, manufactured by WaferTech LLC, decorated with Au nanoparticles by means of aerosol deposition.^44^ The Au nanoparticles were deposited onto the (111)_B_ surface with a nominal diameter of 22 nm at an areal density of 1 particle per μm^2^. The GaAs/InAs core–shell nanowires were grown in a 3 × 2′′ close coupled showerhead metal–organic vapour phase epitaxy (MOVPE) reactor manufactured by AIXTRON GmbH, Germany. The Au-seeded NW growth followed the vapour–liquid–solid (VLS) growth mechanism.^45^ For the GaAs cores trimethylgallium (TMGa) and arsine (AsH_3_), and for the InAs shell trimethylindium (TMIn) and arsine (AsH_3_), were used as the group III and V precursors, respectively. Hydrogen was employed as the carrier gas with a total flow rate of 8 L min^−1^, at a reactor pressure of 100 mbar. After loading the GaAs substrates into the reactor chamber, the samples were annealed for 7 minutes at a set temperature of 645 °C in a H_2_/AsH_3_ atmosphere with an arsine molar fraction of *χ*_AsH_3__ = 2.5 × 10^−3^ to remove oxides from the surface of the samples.

**Fig. 2:**
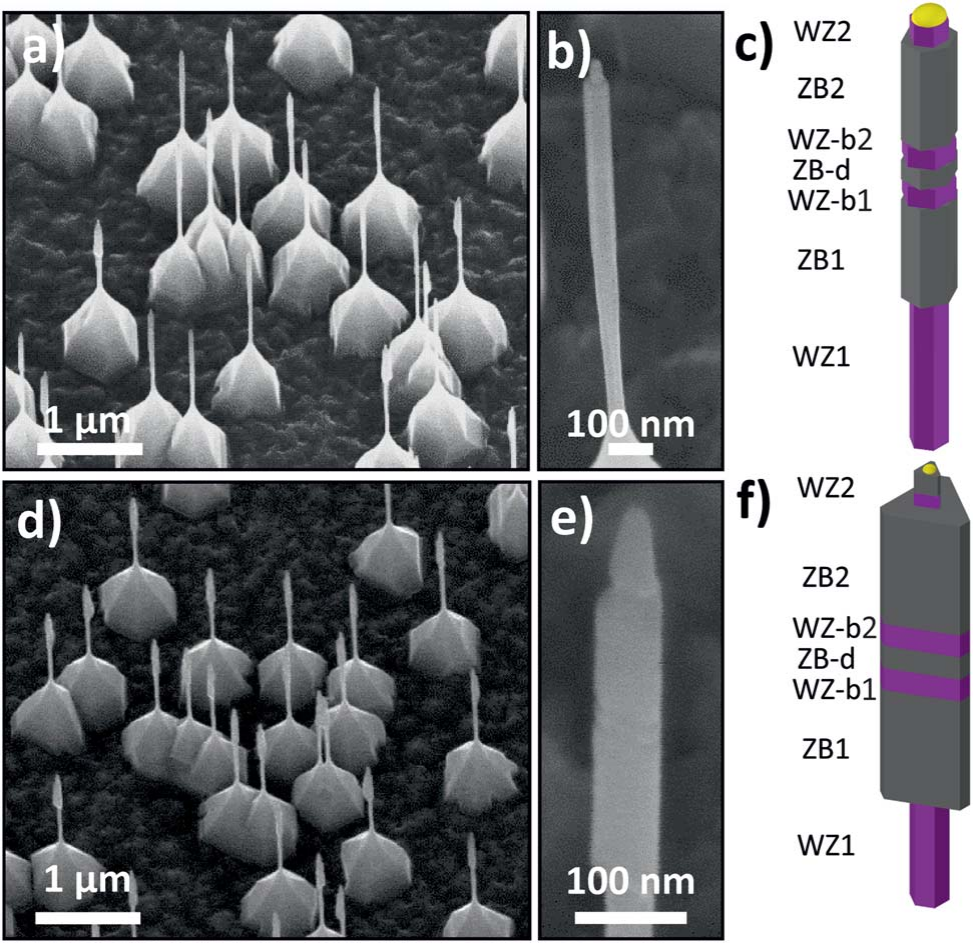
GaAs hexagonal NW cores (a) and (b) 30°-tilted scanning electron micrograph together with (c) a schematic representation of the axial crystal phase encoding. GaAs triangular truncated NW cores (d) and (e) 30°-tilted scanning electron micrograph and (f) schematic representation together with the axial crystal phase encoding. In all micrographs the bottom edge of the figure is aligned with the substrate {110} cleavage edge. For a detailed description of the abbreviations of the different axial nanowire segments given in (c) and (f) please see the paragraph describing the core GaAs nanowire growth.

The growth of the GaAs crystal phase engineered hexagonal core NWs is carried out as follows. After reaching the set growth temperature (540 °C) TMGa and AsH_3_ were supplied simultaneously to initiate GaAs core NW growth with corresponding molar fractions for WZ and ZB of *χ*_TMGa_ = (1.9 × 10^−5^) and *χ*_AsH3_ = (5.0–450 × 10^−5^), respectively. The switching between crystal phases is achieved by changing only the AsH_3_ flow as discussed in ref. 46 and 47. In this case, the conditions previously found to yield WZ and ZB crystal phase are V/III-ratios of 2.4 and 240, respectively.^46,47^ Due to a conduction-band offset between WZ and ZB, in both GaAs^48^ and InAs,^49,50^ the WZ segments confine electrons in the centre ZB segment, resulting in a thin ZB nanodot defined by two WZ barriers. The polytypic sequence of the GaAs hexagonal core NW consists of seven different segments: a first WZ stem (WZ1 – growth time 20 min), followed by a ZB segment (ZB1 – 10 min), a ZB nanodot (ZB-d – 48 s) sandwiched between WZ barriers (WZ-b1 and WZ-b2 – 12 s), another ZB segment (ZB2 – 10–15 min), and a final short WZ segment (WZ2 – 30 s). The final WZ2 segment was grown to reduce the probability of the Au-nanoparticle to slide down from the top part of the NW.^51^ The polytypic sequence is the same for the GaAs hexagonal cross sectional (Fig. 2a to c) and triangular truncated (Fig. 2d and e) cores. In this manuscript we are going to focus on the effects of the InAs shell growth over the GaAs crystal phase engineered cores, being the most interesting part comprised by the ZB1, ZB2, both WZ barriers, and the ZB nanodot. Therefore, the first WZ segment (WZ1) only served as a stem to isolate the NW structure of interest from substrate-related effects. The pyramid observed at the base of the NWs close to the interface with the substrate (Fig. 2a and d) is a result of the vapour–solid (VS) growth^52^ that is taking place simultaneously to the VLS growth.

To investigate the effect of the lateral facet termination on the selectivity of the InAs growth on GaAs, we tune the morphology/facet termination of the cores by homoepitaxially overgrowing them. After this overgrowth step the GaAs NWs cores evolved from hexagonal cross-sectional NWs with {110} sidewalls to triangular truncated NWs with large {112}_B_ lateral facets together with three small ridges, thus, resulting in morphology and lateral facet termination controlled GaAs NW cores. Those ridges can be understood as {112}_A_ or remaining {110} facets (Fig. S1c†). Herein, we will refer to them as ridges. Subsequently the lateral crystal planes of the cores are tuned before exposing them to InAs overgrowth. At 540 °C typical ZB-GaAs NWs grow with {110} lateral facets, however, it is possible to push the development of {112}-type lateral facets by homoepitaxially overgrowing them at a temperature lower than 500 °C.^34,36^ To controllably tune the morphology of the GaAs core NWs, they were overgrown at a set temperature of 450 °C and a nominal V/III-ratio of 2500, with respective molar fractions of *χ*_TMGa_ = 3.7 × 10^−6^ and *χ*_AsH_3__ = 9.4 × 10^−3^ (Fig. 2d to f). The growth time was varied between 5 and 30 min (ESI S1†).

The InAs shells were grown at a substrate set temperature of 450 °C and a nominal V/III-ratio of 132, with respective molar fractions of *χ*_TMIn_ = 1.1–4.5 × 10^−5^ and *χ*_AsH_3__ = 1.5–6 × 10^−3^. The time was varied between 15 s and 15 min and the InAs shells were either grown in one or two steps. For cases when the InAs shells were grown in a 2-step process, the supply of TMIn was stopped after the first step while still supplying AsH_3_, the TMIn flux was adapted, and the supply resumed after precursor flow stabilization to continue with the second growth step. After the final InAs shell growth step the samples were cooled down in an AsH_3_/H_2_ atmosphere.

After the epitaxial growth process, the samples were inspected by means of scanning electron microscopy (SEM) and scanning transmission electron microscopy (STEM) using a SU8010 cold field emission-scanning electron microscope (FE-SEM), Hitachi, Japan, and a LEO 1560 thermal FE-SEM, Zeiss, Germany. Both microscopes were equipped with field emission (FE) guns and operated at 15 kV and 30 kV, respectively. The structural and compositional analysis was carried out by HR-TEM and STEM energy-dispersive X-ray spectroscopy (STEM-EDS) in a JEOL 3000F system, JEOL, Japan, equipped with a FE gun and operated at 300 kV. For TEM analysis, the nanowires were mechanically broken off from the growth substrate and transferred onto lacey carbon covered Cu-grids.

## 3. Results and discussion

### 3.1 Crystal phase selective growth of InAs shells on hexagonal cross sectional GaAs cores

**Fig. 3:**
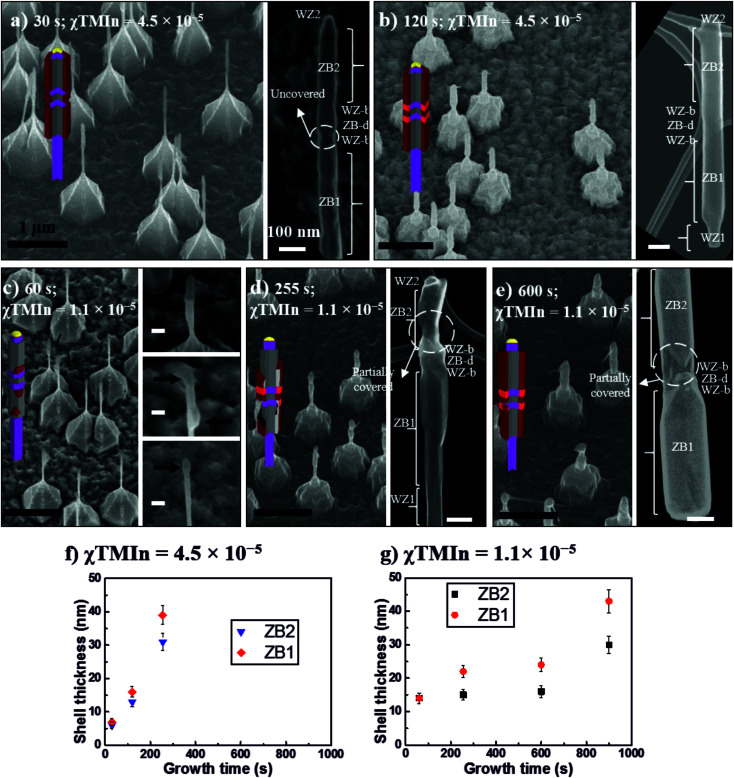
Schematic representation, 30°-tilted SEM micrograph overview, and magnified SEM micrographs of GaAs/InAs core–shell NWs, grown for (a) 30 s at *χ*_TMIn_ = 4.5 × 10^−5^, (b) 120 s at *χ*_TMIn_ = 4.5 × 10^−5^, (c) 60 s at *χ*_TMIn_ = 1.1 × 10^−5^, in the three magnified micrographs the black arrows point to the InAs islands, (d) 255 s at *χ*_TMIn_ = 1.1 × 10^−5^, and (e) 600 s at *χ*_TMIn_ = 1.1 × 10^−5^. Average InAs shell thickness as a function of the growth time for the ZB1 and ZB2 segments for samples grown at a *χ*_TMIn_ of (f) 4.5 × 10^−5^ and (g) 1.1 × 10^−5^. All the GaAs cores follow the crystal phase sequence described in Fig. 1. In all micrographs the bottom edge of the figure is aligned with the substrate {110} cleavage edge. Scale bars are 1 μm (black) and 100 nm (white).

We will first discuss the InAs shell growth over the GaAs hexagonal cores terminated by {110} lateral facets. The morphology of the GaAs/InAs core–shell nanowires is investigated, based on SEM imaging, as a function of the growth time and the TMIn flow rate. In Fig. 3 we show schematic representations along with 30°-tilted overview and magnified SEM micrographs of GaAs/InAs core–shell NWs. In all cases the nominal V/III-ratio was kept constant at a value of 132. Therefore, TMIn and AsH_3_ flows were changed to maintain the stated V/III-ratio. Consequently, the total precursor supply and, thus, the nominal growth rate were higher for higher TMIn flows (Fig. 3a and b). For the sake of simplicity, the growth conditions with a *χ*_TMIn_ of 4.5 × 10^−5^ (Fig. 3a and b) and 1.1 × 10^−5^ (Fig. 3c to e) will be referred to in the following as nominally high and low growth rate, respectively.

The shells grown at a nominally high growth rate (Fig. 3a and b) show a homogeneous InAs coverage of the core NW. For 30 s growth time (Fig. 3a) the nucleation of the InAs shell takes place preferentially over the long ZB segments (ZB1 and ZB2), while being significantly hindered over the WZ segments. The high growth rate conditions potentially lead to the appearance of a significant amount of stable nuclei that grow laterally until they eventually coalesce, thus, resulting in a continuous shell coverage over the ZB regions after few seconds of InAs growth. A similar behaviour has been observed for InAs on GaAs,^40,53^ and GaSb on InAs.^41,42^ After 120 s growth time (Fig. 3b) the InAs shell has not only grown radially over the ZB-GaAs segments, but also covered the WZ-GaAs. The growth over the WZ crystal phase commences later, most likely initiated at the ZB segments and expanded over the WZ segments.

In contrast, when discussing the results of shells grown at a nominally low growth rate (Fig. 3c–e) we observe a non-homogeneous InAs shell coverage. This inhomogeneity can be divided into two steps. First, we observe an inhomogeneity of the initial InAs shell formation, which is highlighted in the magnified SEM micrographs of Fig. 3c, where 3 different NWs show islands nucleated at different positions of the GaAs cores and occurs predominantly at the ZB segments. Secondly, it can be noted from the high-resolution SEM micrographs (Fig. 3d and e), that the InAs shell, after coalescence on the ZB segments, is not covering the cores homogeneously. In particular, the ZB nanodot and both WZ barriers are not fully covered by the InAs shell. Both observations indicate a crystal phase selective nucleation and growth, which can be seen especially for longer growth times (Fig. 3d and e). Ultimately, a full shell is formed after even longer growth times of 900 s (not shown) similar to the case of high growth rate at 120 s with similar shell thicknesses but at an overall lower level of homogeneity.

It is further noticeable that the Au-alloy nanoparticle (NP) has been displaced from the top WZ2 segments of most NWs (Fig. 3b to e), since the InAs growth conditions are chosen to favour the radial over the axial growth. Under these growth conditions the NP most likely becomes unstable on the WZ(0001̄) top facet and the NP slides down until it reaches the interface with the ZB2 segment. Tornberg and co-workers demonstrated that a ZB insertion can act as a potential barrier in order to prevent the NP from sliding down further on the NW side facets.^51^ Then, the NP stops and starts branched growth away from the NW core similar to some cases visible in the overview SEM images of Fig. 3b, d and e.

The average thickness of the InAs shells over the ZB1 and ZB2 segments is presented in Fig. 3f and g as a function of the growth time at TMIn molar fractions of 4.5 × 10^−5^ and 1.1 × 10^−5^, respectively. Shell thicknesses are extracted from STEM micrographs taken in an SEM by comparing the thickness of the core–shell NW with the thickness of the bare GaAs cores (Fig. 2a). In the case of the InAs shells grown at a high growth rate the thickness increases linearly with growth time. In contrast, for the shells grown at a low growth rate the increase is not linear until a growth time of about 600 s is reached. This difference can be attributed to the initial island growth mode found for the shells grown at a low growth rate. The shells first seem to grow laterally until they coalesce and then a linear behaviour of the InAs shell thickness *vs.* growth time is expected and can be noticed from Fig. 3g.

For high and low growth rate the InAs coverage of the ZB1 segment (bottom) is found to be higher than on the ZB2 segment (top). In addition, this difference is larger for shells grown at low growth rate than for shells formed under high growth rate conditions. For both cases presented above the first points (30 s and 60 s, respectively) are excluded as a non-uniform coverage was observed there. The general observation of a higher thickness on ZB1 compared to ZB2 segments could be attributed to an additional material supply provided by collection on the substrate and/or WZ stem. The average thickness of a fully coalesced shell is 16 and 13 nm at high growth rate for ZB1 and ZB2, respectively, and 43 and 30 nm (ZB1 and ZB2) at low growth rate. This finding is a strong indication that a higher overall degree of control can be achieved for the high growth rate case.

**Fig. 4:**
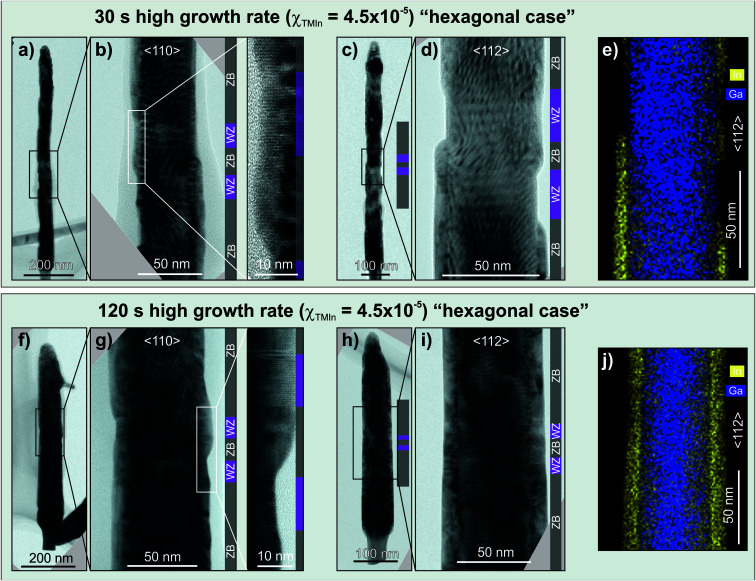
TEM bright field (BF) micrographs and STEM-EDS maps of GaAs core NWs with an InAs shell grown at a high growth rate for 30 s (a)–(e) and 120 s (f)–(j), respectively, and taken in either 〈110〉 (a), (b), (f) and (g) or 〈112〉 (c)–(e) and (h)–(j) direction. A smooth and continuous shell is formed after 120 s of growth (f)–(j) while it is incomplete after 30 s (a)–(e) with indications for preferential coverage of the ZB segments rather than the WZ segments. WZ (purple) and ZB (grey) segments are indicated by additional colour bars in the BF-TEM micrographs.

The TEM analysis of NWs from samples grown for 30 s and 120 s at a high growth rate is presented in Fig. 4. For the InAs shells grown for 30 s a TEM bright field (BF) overview image, taken along the 〈110〉 zone axis, is shown on Fig. 4a and, with a higher magnification, in Fig. 4b. It reveals the stacking sequence of the NW which is additionally highlighted by a color-coded bar demonstrating that the crystal structure transition at the axial heterointerfaces between the WZ and ZB and *vice versa* is sharp. It is worth mentioning that the exact position of the interface between the GaAs core and the InAs shell cannot be determined from micrographs taken along a 〈110〉 zone axis, as the NWs have {110} side facets and therefore the imaging direction is along an edge rather than a flat facet. Therefore, a TEM BF micrograph of a different NW taken along a 〈112〉 zone axis is shown in Fig. 4c and d together with a STEM-EDS map of In- and Ga-related signals of the identical NW region (Fig. 4e). It indicates the radial interface of the GaAs/InAs core–shell structure demonstrating the uneven shell formation mainly caused by the partially suppressed InAs growth over the WZ barrier region. It can be further noticed on the right side of Fig. 4d that the growth of the shell is reduced over the top WZ barrier and ZB nanodot while on the left side the InAs shell covers the bottom WZ barrier and the ZB nanodot. A potential explanation for this observation is that the growth over the WZ phase is advancing from the ZB segment. It is worth mentioning the apparent but non-uniform crystal phase selectivity of the InAs shell growth which is obvious from the STEM-EDS map (Fig. 4e). This non-uniform selectivity might be attributed to a slightly too long growth time that results in the formation of a complete InAs shell.

Fig. 4f to j present the TEM analysis of the InAs shells grown for 120 s at a high growth rate, showing the thickness distribution of the shell along the NW core. Therefore, this demonstrates a homogenous, non-crystal phase selective growth mechanism for a longer deposition time.

In order to explain the observed crystal phase selectivity at shorter growth times, it should be taken into account that ZB surfaces usually possess higher surface energies than their comparable WZ counterparts.^46,54^ Therefore, at certain growth conditions, it is possible to intentionally hinder or even suppress the growth over the WZ segments, while favouring it on the ZB phase. In our case the GaAs WZ segments have {10−10}- and/or {11−20}-type lateral facets whose surface energies are predicted to be lower than the {110}-type facets on the corresponding GaAs ZB segments^55^ and, thus, supporting the claim phrased above. However, it should be noted here that different lateral facets can have different surface energies and, consequently, the observed selectivity may be different, opposite or even absent, for other facet and/or material combinations.

A low growth rate results in adatom incorporation into the crystal at thermodynamically favourable sites due to a larger adatom diffusion length. This leads to the nucleation and growth of InAs islands (Fig. 3c) in order to reduce the accumulated strain energy.^56^ As stated previously, the difference in surface energy for WZ and ZB facets affects the nucleation probability of InAs on GaAs. As the growth proceeds, further atom incorporation will take place preferentially on the already nucleated InAs islands, favoured by the growth conditions. Consequently, these islands will continue growing axially and radially until they eventually coalesce forming a closed InAs shell on first the ZB segments and secondly the WZ segments. Therefore, this growth mechanism initially seems to be sensitive to the crystal phase but the level of homogeneity of the shell formation in terms of thickness and coverage is very poor.

A high growth rate leads to a lower adatom diffusion length which in turn leads to a predominately kinetically limited regime, where adatoms can only diffuse short distances before they are incorporated into the crystal or desorb from the surface. Therefore, island formation is kinetically delayed or frozen out and the shell grows in a kinetically limited regime. In addition, from the obtained selectivity (Fig. 4a to e) a lower nucleation barrier for InAs on ZB-GaAs than on WZ-GaAs can be inferred as well. For a short growth time of 30 s or less (Fig. 4a to e) InAs nucleates preferentially on ZB-GaAs, however, it also nucleates on WZ-GaAs but at a much lower rate. Therefore, the growth is crystal phase selective in the initial stages. However, for long enough growth times (*e.g.* 120 s, Fig. 4f to j) substantial InAs growth occurs on WZ GaAs and the growth is no longer crystal phase selective.

Thus, according to the interpretation of our growth results, it is possible to enable a crystal phase selective GaAs/InAs core–shell growth by mainly adjusting the two key growth parameters, namely choosing a high growth rate at a short growth time.

### 3.2 Facet dependent selective InAs growth on triangular truncated GaAs cores

To study the facet dependent selective growth of InAs on GaAs, we grew a set of InAs shells (Fig. S2†) with varying deposition times onto triangular truncated GaAs cores. For more information on the formation of triangular truncated GaAs cores by means of homoepitaxial overgrowth of GaAs, the reader is kindly referred to the ESI, Sections S1 and S2.† The InAs shell growth conditions correspond to the ones defined as the nominally high growth rate (please see Section 3.1 for more details).

**Fig. 5:**
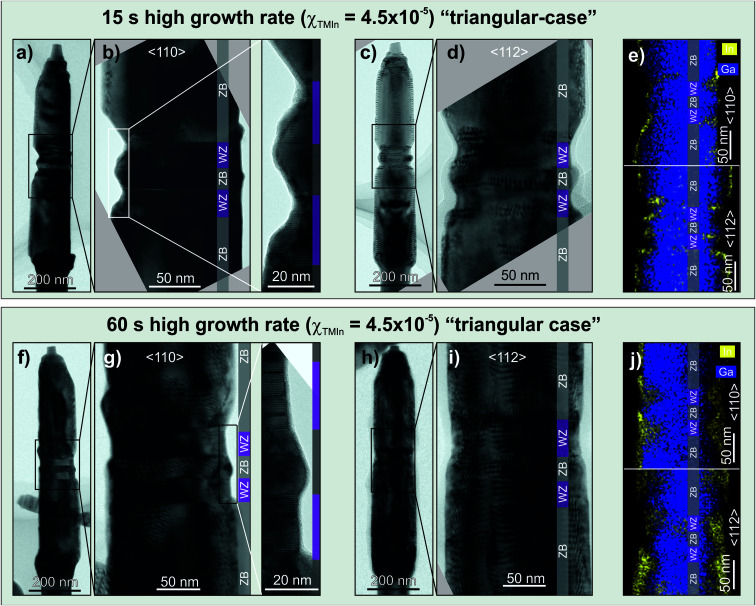
TEM bright field (BF) micrographs and STEM-EDS maps of GaAs core NWs with an InAs shell grown at a high growth rate for 15 s (a)–(e) and 60 s (f)–(j), respectively, and taken along the 〈110〉 (a), (b), (f) and (g) or 〈112〉 (c), (d) and (h), (i) zone axis. STEM-EDS colour maps (e) and (j) reveal that the InAs shells grow preferentially around the dot region probably due to the zig–zag profile of the core. WZ (purple) and ZB (grey) segments are indicated by additional colour bars in the BF-TEM micrographs.

NWs from the samples with InAs shells grown for 15 s and 60 s were analysed by TEM (Fig. 5). As the NW core has a triangular truncated morphology with {112}-type side facets it is possible to determine the exact position of the interface between the GaAs core and the InAs shells.

During the homoepitaxial overgrowth the radial growth rates of the ZB and WZ crystal phases are different. This is particularly relevant for the ZB nanodot and WZ barrier regions (left side of Fig. 5b, and both sides of Fig. 5d) where the morphology resembles to a zig–zag profile due to a difference in thickness and lateral facet termination. The ZB nanodot presents {111}-type inclined side facets and not {110} ones as in the hexagonal case. In addition, the WZ barriers are smaller in diameter than the surrounding ZB sections. Therefore, those inclined facets together with the smaller diameter of the WZ barriers create the mentioned zig–zag morphology in the ZB nanodot and WZ barriers region. The chemical analysis is presented on Fig. 5e by means of a STEM-EDS colour map along both zone axes, 〈110〉 and 〈112〉. On the colour map taken along the 〈110〉 direction a preferential accumulation of InAs on the ridges can be appreciated. The colour map taken along the 〈112〉 direction confirms the preferential accumulation of InAs on the edges of the NW, corresponding to the {112}-type lateral facets, together with indications of a lower coverage on the WZ segments pointing towards crystal phase selectivity in that case as well.

For the InAs shells grown for 60 s over a triangular truncated GaAs core (Fig. 5f to j), the Moirè fringes are distributed all over the NW being more prominent over the ridges. This can be related with a higher accumulation of InAs on them. This interpretation is indeed confirmed by both STEM-EDS colour maps (Fig. 5j). A significant accumulation of InAs can be found in the ZB-dot region, most likely due to the zig–zag morphology of the GaAs core described previously. An even longer growth time (>120 s) results in a full InAs shell formed over the complete GaAs core (Fig. S2†).

In this case, the mechanism regarding this selectivity is different than the one presented in Section 3.1 for the hexagonal GaAs cores with {110} side facets. The mentioned zig–zag profile of the GaAs core, around the ZB-dot region, modifies the local conditions during the growth of the InAs shell, creating areas prone to the nucleation of InAs. Once the nucleation has started, the In and As atoms arriving to the ZB nanodot and WZ barriers region have a higher probability of being incorporated at that region. Therefore, the growth in the ZB nanodot and WZ barriers region is taking place in a kinetically limited regime. The growth of InAs starts confined to the ridges as demonstrated in Fig. S2b and c.† As the growth proceeds, the InAs is not only expanding towards the WZ phase, but also along the ZB {112}_B_ facets, growing radially perpendicular to the ridge facets and along the 〈110〉 direction until the {112}_B_ facet is also covered by InAs. Therefore, the growth rate of InAs over the {112}_B_ facet is lower than over the ridges, but still has to be taken into account. Talking about the InAs growth confined to the ridges, the strain energy minimization plays an important role in driving the InAs to adopt the observed selectivity. Therefore, growing on a ridge-like structure will help in relieving the stress which is accumulated due to the lattice mismatch between GaAs and InAs. This preferential growth path through efficient stress relaxation has been studied *e.g.* for GaAs/InAs NW networks.^35^

It is worth mentioning that the effect of crystal phase selective shell growth might be suppressed by the InAs shell growth in a kinetically limited regime around the zig–zag region of the core. To get a clearer vision of the facet dependent and crystal phase selective growth, decoupling of both effects is desired. To ultimately do so, we propose an additional step during the homoepitaxial overgrowth of the GaAs core NW in a kinetically limited regime to enhance the growth over the ZB nanodot region and thus reducing the effect of the zig–zag morphology on the shell growth. However, the development of such “zig–zag-free” core morphologies is beyond the scope of this work.

### 3.3 Two-step InAs shell growth on GaAs cores

In view of the shell growth evolution observed for both cases, hexagonal and triangular, there is a selective growth in the initial stage of the growth process for the growth conditions employed. This can be either a crystal phase, a facet dependent selective growth, or both. However, the selective growth is not preserved for longer growth times and the thickness of the InAs shell grown in the crystal phase selective conditions is limited to the initial stages of the shell growth. Likewise, for the triangular truncated case, the dimensions of the InAs nanowires grown on the {112}_A_-like ridge facets are determined by the size of the ridge, and the InAs shell growth time. In order to maintain the InAs segments isolated from each other it is necessary to limit the growth time. The natural question arising from these findings is whether it is possible to grow a thick InAs shell without losing the achieved selectivity, *i.e.*, keeping it confined to the ZB crystal phase, to the ridges in triangular NWs, or both.

**Fig. 6:**
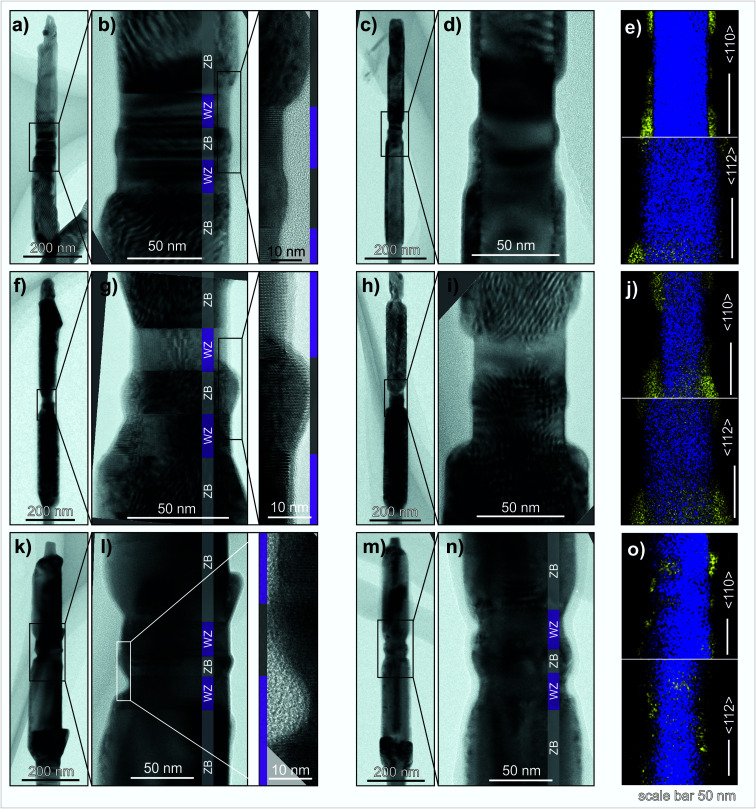
TEM bright field (BF) micrographs and TEM-EDS maps of GaAs core NWs with an InAs shell grown in 2-steps at a high and low growth rate on hexagonal cores for (a)–(e) 15 s and 30 s, (f)–(j) 15 s and 255 s and (k)–(o) on triangular cores for 15 s and 30 s, respectively. The micrographs were taken in either 〈110〉 (a), (b), (f), (g), (k) and (l) or 〈112〉 (c), (d), (h), (i), (m) and (n) direction, whereas the STEM-EDS colour maps, (g), (k) and (l), were collected along both directions. WZ (purple) and ZB (grey) segments are indicated by additional colour bars in the BF-TEM micrographs.

To do so we design a 2-step InAs growth approach which was tested both on the hexagonal and triangular truncated GaAs NW cores. The InAs 2-step growth is comprised by an initial first InAs step grown at a nominally high growth rate followed by a second step at a nominally low growth rate. The growth temperature and the V/III-ratio were kept constant, for both steps, at 450 °C and 132, respectively. Three different samples were analysed by TEM and are presented in Fig. 6. Two of them were grown on hexagonal GaAs cores (Fig. 6a to j) and the third one was grown on a triangular truncated GaAs core (Fig. 6k to o). More information on the choice of the growth times can be found in Section S3 of the ESI.†

Fig. 6a to e give TEM data of the InAs shell grown for 15 s and 30 s for the first and second step, respectively. The GaAs/InAs core–shell interface is shown on the micrographs taken along the 〈112〉 zone axis (Fig. 6c and d).

It can be appreciated that on the right side of Fig. 6d the shell grows selectively over the ZB segments and dot whereas on the left side of the NW, the InAs shell additionally covers the bottom WZ barrier. This might have occurred since the growth over the WZ phase is advancing from the ZB segment, and the growth time was chosen slightly too long and could be avoided by a more appropriate growth time management.

The compositional analysis is presented on Fig. 6e by means of a STEM-EDS colour map. It shows that the InAs shell growth is taking place preferentially over the ZB segments and is reduced over the WZ barriers. So, for a growth time of 15 s and 30 s for first and second steps, respectively, the crystal phase selectivity is kept. However, to validate the 2-step approach, a shell grown with a longer second step is mandatory.

The next NW sample, Fig. 6f to j, corresponds to the InAs shell grown on a hexagonal core for 15 s and 255 s for the first and second step, respectively. The magnified TEM BF micrograph (Fig. 6i taken along the 〈112〉 zone axis) demonstrates that the shell grows preferentially over the ZB segments. Therefore, the crystal phase selectivity is preserved for a longer deposition time of step 2. A STEM-EDS colour map (Fig. 6j) addresses the chemical composition of the core–shell NW. Both maps, along the 〈110〉 and 〈112〉 directions, show an area where the In signal is reduced, corresponding to the WZ barriers and ZB nanodot region. In addition, Moirè fringes together with the EDS maps give indications for enhanced InAs nucleation and growth on the ZB-dot region compared to the WZ barrier segments. All together this indeed demonstrates that the achieved crystal phase selectivity can be maintained for longer deposition times using the 2-step approach.

The TEM analysis of the third NW sample, grown on a triangular truncated GaAs core NW, is presented on Fig. 6k to o. The InAs shell was grown for 15 and 30 s, first and second steps, respectively. The core–shell interface is depicted on Fig. 6k and l. On the right edge of the STEM-EDS colour map taken along the 〈110〉 direction, an accumulation of InAs on the ZB segments and ZB nanodot can be appreciated, but far less on the WZ barriers. Whereas for the left side, where the zig–zag profile is more prominent, InAs accumulates on the ZB segments and dot, and partially on the WZ barriers as well. As stated in the previous section, the effect of crystal phase selectivity might be suppressed or hindered by the zig–zag morphology of the GaAs core resulting in the InAs growth in a kinetically limited regime.

The success of the 2-step growth of InAs shells, especially over the hexagonal cores, can be attributed to the combination of the two growth regimes presented in Section 3.1. In the case of hexagonal cores, as we have demonstrated, the high growth rate regime first leads to a preferential growth over the ZB regions. As this growth regime proceeds, the InAs shell grows from the ZB towards the WZ phase and for long enough growth times the entire NW can be covered with a homogeneous shell. With the 2-step growth instead, we limit the time of the high growth rate step to preserve the crystal phase selectivity. During the second step, the growth rate is reduced, resulting in an increase of the adatom diffusion length that allows the arriving In and As atoms to incorporate into the crystal at thermodynamically favourable sites. Furthermore, taking into account the non-negligible lattice mismatch between GaAs and InAs (∼7% for ZB(100) and on a similar level for the other occurring facets), the incorporation of incoming In and As atoms into the crystal will take place preferentially on the already formed InAs shell. It is energetically favourable for the In and As atoms to be incorporated into the already grown shell rather than to nucleate a new island directly on the GaAs surface. Consequently, the radial shell growth (over the ZB phase) is favoured over the axial growth (over the WZ phase). However, the growth over the WZ phase is not completely suppressed as it can be appreciated on Fig. 6j, where a weak In signal can be observed over the WZ phase which could be a result of a slightly too long step 1 or by the fact that the crystal phase selectivity might be lost for a very long second step. However, that situation will occur only if there is a non-negligible kinetic contribution to the nucleation and/or growth over the WZ segments.

For the triangular truncated case, the scenario is more complex. As mentioned above, the first step promotes the selective growth, and the second step allows the incoming In and As atoms to be incorporated on thermodynamically favourable sites. In addition to the different crystal phases present, we need to add the contribution from the different crystal facets and the zig–zag morphology around the dot region. In Fig. 6o, zone axis 〈110〉, the effect of the zig–zag morphology on the selectivity can be appreciated, where the InAs grows on the WZ barriers and not on the ZB nanodot. Whereas on the right side of the GaAs core InAs grows on the ZB segments and the nanodot. So, the zig–zag morphology has an influence on the selectivity that should be considered and crystal-phase selectivity would be more apparent for flat side facet morphology rather than the zig–zag morphology. However, the development of such core morphologies is beyond the scope of the presented study.

## 4. Conclusions

Summarizing, we demonstrate a two-fold selectivity control which is driven by the different crystal phase (WZ or ZB), by the different lateral facet termination present in the NW core, or both. In addition, we have determined the two key growth parameters, growth rate and growth time, governing the selective core–shell growth. The crystal phase selectivity was studied on GaAs NW cores with controlled crystal phase in the axial direction. A crystal phase selective growth of InAs on GaAs was found for high growth rates and short growth times. However, selectivity was lost after longer growth times. The selectivity against facet termination was studied employing morphology tuned GaAs cores, where the GaAs NW cores evolved from hexagonal NWs with {110} sidewalls to triangular truncated NWs with 3 {112}_B_ facets and 3 ridges. By controlling the growth conditions, the growth of InAs is promoted over the ridges and reduced over the {112}_B_ facets, again, for short growth times. The preferential growth over the ridges can be partially attributed to the more effective strain relaxation of the InAs shell over them. According to our interpretation of the results, the effect of the crystal phase selectivity is less pronounced by a kinetically limited growth due to the zig–zag morphology of the core around the ZB nanodot region. The crystal phase as well as the facet dependent selectivity are lost for long enough growth times, thus, limiting the total thickness of the InAs shell grown in conditions stimulating the selectivity. Finally, we propose a 2-step growth approach to control the thickness of the InAs shells, formed on crystal phase engineered GaAs cores, while keeping the selectivity of the growth. We demonstrate, by means of a detailed HR-TEM analysis, that the 2-step growth is a promising route to control the thickness of the InAs shells while keeping the selective growth and thus paves the way to designing highly complex, three-dimensional InAs structures and can potentially be expanded to other material combinations as well.

## Conflicts of interest

The authors declare that there are no conflicts of interest.

## Supplementary Material

NA-004-D2NA00109H-s001
