# Supplementary material for: Growth selectivity control of InAs shells on crystal phase engineered GaAs nanowires
Source: Nanoscale Adv. 2022 Apr 8;4(16):3330–41. doi: 10.1039/d2na00109h (PMC9417278; doi:10.1039/d2na00109h)
Supplement: NA-004-D2NA00109H-s001 [file NA-004-D2NA00109H-s001.pdf]

Electronic Supplementary Material (ESI) for Nanoscale Advances.
This journal is © The Royal Society of Chemistry 2022

Supplementary material to:

Growth selectivity control of InAs shells on crystal phase engineered GaAs nanowires

Víctor Jesús Gómez*[,a,b], Mikelis Marnauza[c], Kimberly A. Dick[b,c], and Sebastian Lehmann[b]

[a] *Nanophotonics Technology Center, Universidad Politécnica de Valencia, Camino de Vera, s/n Building 8F | 2ª Floor, 46022 Valencia, Spain*

[b] *Solid State Physics and NanoLund, Lund University, Box 118, S-221 00 Lund, Sweden.*

[c] *Centre for Analysis and Synthesis, Lund University, Box 124, 221 00, Lund, Sweden.*

S1 **GaAs core morphology tuning** **page 2**

S2 **InAs shell growth on triangular truncated GaAs NW cores:**
Time series for high growth rate **page 4**

S3 **2-step InAs growth on triangular truncated GaAs cores** **page 5**

S1. GaAs core morphology tuning

We tune the morphology of the GaAs cores, and subsequently the exposed lateral crystal plane, by homoepitaxial overgrowth. Figure S1 shows the time evolution of the morphology of the cores during homoepitaxial GaAs overgrowth which was carried out at the conditions described in the experimental section 2 (T_{OG} = 450 °C, and V/III = 2500) [1,2]. After 30 minutes of homoepitaxial overgrowth, the nanowires (NWs) exhibit a triangular cross sectional shape with only three $\{112\}_B$ lateral facets (Fig. S1 d).

Alternating ZB segments can be twinned with respect to each other due to the insertion of WZ segments in between them although the ZB segments itself are free of twin defects. This twinning is responsible for the rotation between different ZB segments as shown exemplarily in Fig. S1 e) and leads to theoretically only 25% of the NWs having all the ZB segments fully aligned, a number that roughly corresponds to our observations.

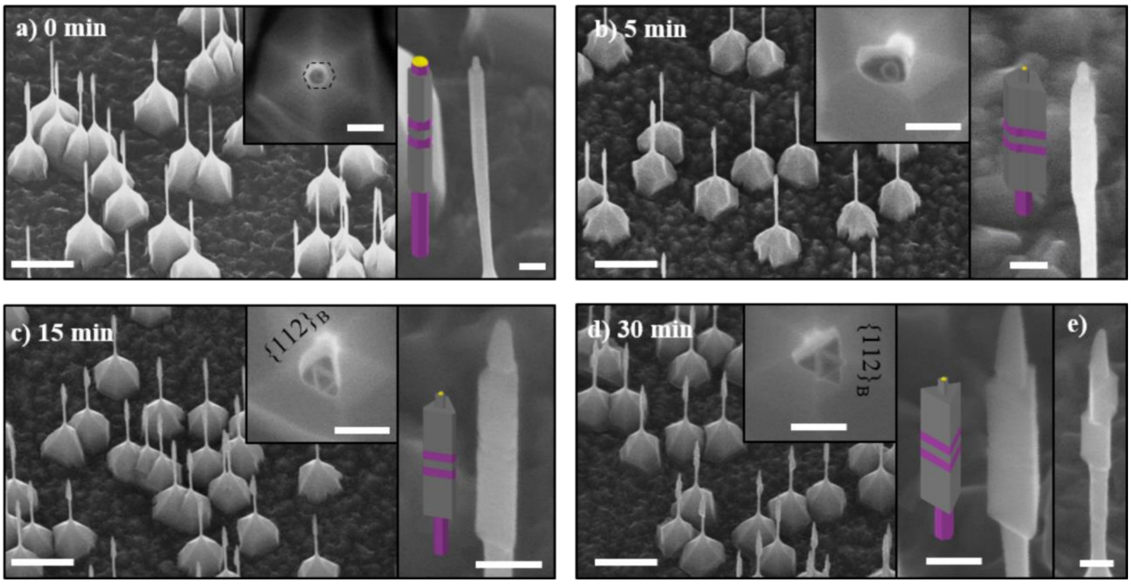

Fig. S1 30°-tilted overview and magnified top view and 30°-tilted SEM micrographs (insets) together with the schematic representations of GaAs core NWs homoepitaxially overgrown for a) 0 minutes, b) 5 minutes, c) 15 minutes, and d) 30 minutes. e) 30°-tilted SEM micrograph of a single NW from sample d) where the ZB segments are twinned with respect to each other. In all micrographs the bottom edge of the figure is aligned to the substrate {110} cleavage edge. Scale bars lengths are 1μm in the overview SEM images and 100 nm in all insets.

S2. InAs shell growth on triangular truncated GaAs NW cores: time series for high growth rate

Figure S2 a shows the triangular truncated GaAs NW core as a reference. After 15 s of InAs overgrowth (Fig. S2 b) InAs nucleates preferentially on the ridges while being hindered over the $\{112\}_B$ facets. After 60 s of growth (Fig. S2 c) a clear InAs shell is visible at the position of the truncated corner facet. In addition, InAs growth partially starts spreading over the $\{112\}_B$ facets. For a longer growth time (120 s, Fig. S2 d), the InAs continues growing radially, perpendicular to the $\{112\}_A$ facet, and along the <110> direction until the WZ segments and the $\{112\}_B$ facets are also covered. The latter observation agrees with the work reported by Paladugu and co-workers [3]. It is worth mentioning that the growth of InAs takes place radially as well as axially. The latter can be noticed e.g. in Figure S2 b, c, and d where an InAs segment has formed on top of the WZ2 segment. In some NWs, especially in Figure S2 c and d, this axial InAs stem is kinked. This mechanism is explained in more detail in section 3.1 in the main text.

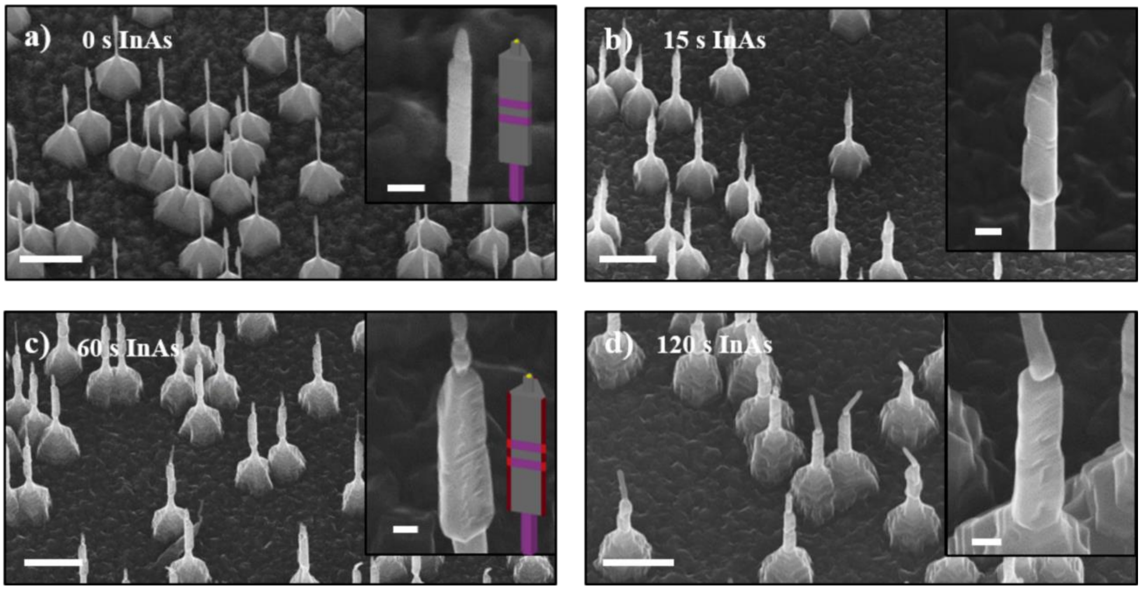

Fig. S2 30°-tilted SEM micrographs of InAs shells grown on GaAs triangular truncated NW cores for a) 0 s, b) 15 s, c) 60 s, and d) 120 s. On every figure, the insets show a close-up micrograph of a single GaAs/InAs NW. In all micrographs the bottom edge of the figure is aligned to the substrate {110} cleavage edge. Scale bars lengths are 1 μm in the overview SEM images and 100 nm in all insets.

S3. 2-step InAs shell growth on triangular truncated GaAs NW cores

Figure S3 shows 30°-tilted SEM micrographs of the GaAs/InAs NWs grown using the 2-step approach as described in detail in section 3.3. The growth time for the first and second step were 5 and 15 s, and 30 and 120 s, respectively. In these cases, a pronounced InAs accumulation is found at the bottom of ZB1 segment marked with a white arrow in the magnified micrographs (Fig. S3). This InAs accumulation was already noticeable after the first InAs growth step as presented in the previous section (Fig. S3 b). However, this accumulation is significantly larger after the two-step growth, being enhanced during the low growth rate step.

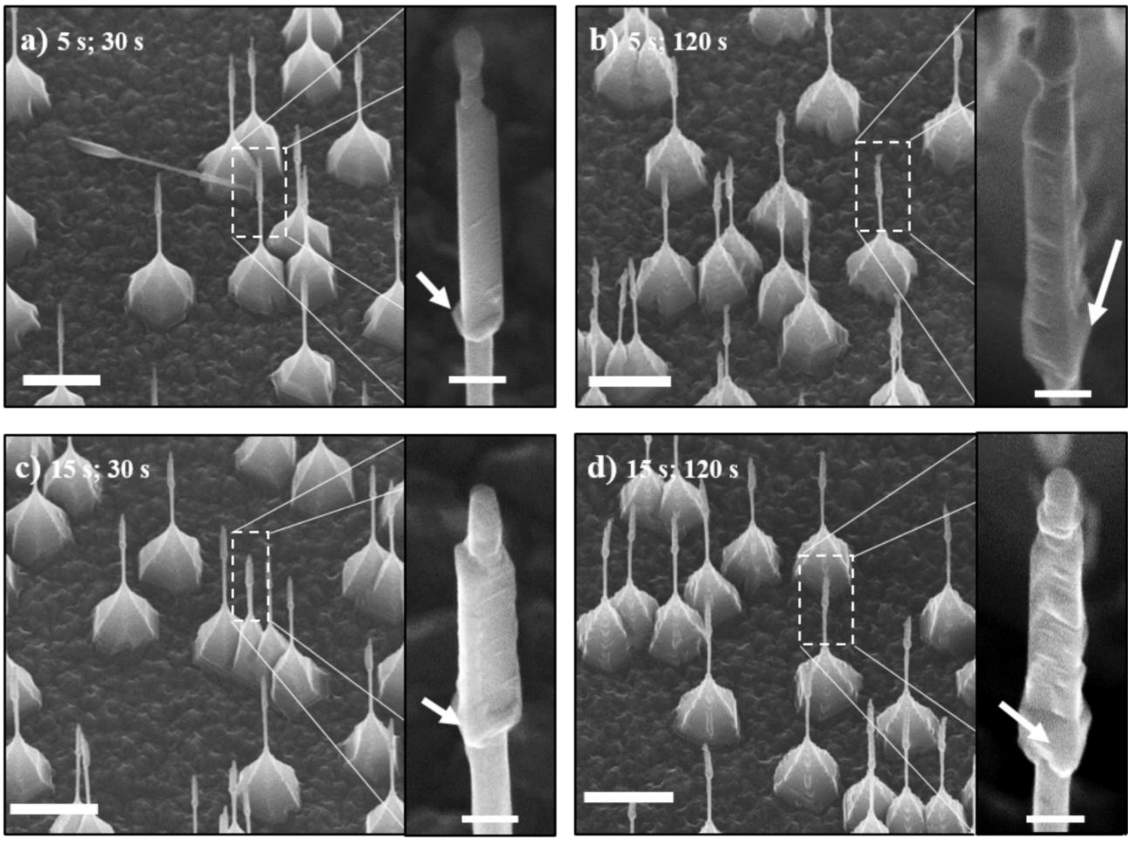

Fig. S3 30°-tilted overview and magnified SEM micrographs (insets) of the 2-step InAs shell growth on triangular truncated GaAs core NWs, for a) 5 and 30 s, b) 5 and 120 s, c) 15 and 30 s, and d) 15 and 120 s, where the first and the second times correspond to the growth times of the first and the second step, respectively. The white arrows point towards the InAs accumulation at the bottom of the first ZB segment. In all micrographs the bottom edge of the figure is aligned to the substrate {110} cleavage edge. Scale bars lengths are 1μm in the overview SEM images and 100 nm in all insets.

References:

[1] Jiang N, Wong-Leung J, Joyce H J, Gao Q, Tan H H and Jagadish C 2014 Understanding the True Shape of Au-Catalyzed GaAs Nanowires *Nano Lett.* **14** 5865–72

[2] Zou J, Paladugu M, Wang H, Auchterlonie G J, Guo Y-N, Kim Y, Gao Q, Joyce H J, Tan H H and Jagadish C 2007 Growth Mechanism of Truncated Triangular III–V Nanowires *Small* **3** 389–93

[3] Paladugu M, Zou J, Guo Y-N N, Zhang X, Kim Y, Joyce H J, Gao Q, Tan H H and Jagadish C 2008 Polarity driven formation of InAs/GaAs hierarchical nanowire heterostructures *Applied Physics Letters* **93** 101911
